# Voluntary Slow Breathing Exercise on Cardiovascular Parameters in Patients With Hypertension: A Systematic Review and Meta‐Analysis

**DOI:** 10.1002/clc.70279

**Published:** 2026-03-25

**Authors:** ShenTing Cheng, Yan Chen, Man Yu, LiShuang Zhao, Hui Huang, MinYan Zhao

**Affiliations:** ^1^ The Second Affiliated Hospital of Soochow University Suzhou City Jiangsu Province China; ^2^ Soochow University Suzhou City Jiangsu Province China; ^3^ Department of Nursing The Second Affiliated Hospital of Soochow University Suzhou City Jiangsu Province China

**Keywords:** blood pressure, breathing exercise, heart rate, hypertension, slow breathing

## Abstract

**Background:**

Non‐pharmacological interventions are increasingly recognized as complementary approaches in the management of hypertension. Among various non‐pharmacological interventions, voluntary slow breathing exercise has been proposed as a simple and accessible approach, but its comprehensive effects on cardiovascular parameters in hypertensive patients remain to be systematically summarized.

**Aim:**

This systematic review and meta‐analysis aims to quantitatively assess the effects of voluntary slow breathing exercise on cardiovascular parameters in individuals with hypertension.

**Methods:**

Following the Preferred Reporting Items for Systematic Reviews and Meta‐Analyses (PRISMA) guidelines, we conducted literature searches in four major databases (PubMed, Web of Science, Embase, and the Cochrane Library) to identify eligible studies. Meta‐analysis was conducted using RevMan 5.4 software. Effect sizes were pooled by calculating mean differences (MDs) and their 95% confidence intervals (CIs). A fixed‐effects or random‐effects model was used according to the results of the heterogeneity test.

**Results:**

A total of thirteen studies met the predefined inclusion criteria. The pooled analysis demonstrated that voluntary slow breathing exercise significantly reduced systolic blood pressure (MD = −7.68; 95% CI: −9.87, −5.49; *p* < 0.001), diastolic blood pressure (MD = −4.02; 95% CI: −5.44, −2.60; *p* < 0.001), and heart rate (MD = −1.15; 95% CI: −1.73, −0.56; *p* < 0.001) compared to control conditions. Additionally, a significant reduction in the low‐frequency to high‐frequency power ratio (LF/HF) was observed (MD = −1.09; 95% CI: −1.94, −0.23; *p* = 0.01), indicating improved autonomic modulation.

**Conclusion:**

Voluntary slow breathing exercise appears to be an effective adjuvant non‐pharmacological intervention for improving cardiovascular function, particularly in reducing blood pressure and heart rate, in patients with hypertension. These findings support its potential integration into hypertension management strategies.

## Graphical abstract

Voluntary slow breathing exercise lowers systolic (−7.68 mmHg), diastolic blood pressure (−4.02 mmHg), and heart rate (−1.15 bpm), based on 13 RCTs including 1,097 participants.

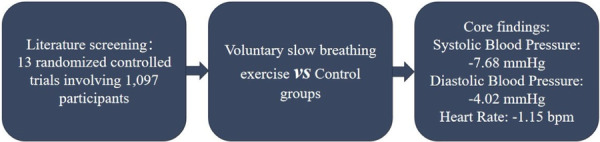

## 1 Introduction

Hypertension, defined as a systolic blood pressure ≥ 140 mmHg and a diastolic blood pressure ≥ 90 mmHg or use of antihypertensive medication, can lead to severe ailments such as stroke, myocardial infarction, dementia, and renal diseases if poorly controlled [1, 2, 3, 4]. It is the leading cause of cardiovascular disease and premature death worldwide [5], accounting for 10.8 million deaths worldwide. Hypertension affects 1 in 3 adults worldwide. The number of people living with hypertension (blood pressure of 140/90 mmHg or higher or taking medication for hypertension) doubled between 1990 and 2019, from 650 million to 1.3 billion [6]. For adults with hypertension and prehypertension, early use of antihypertensive medications and lifestyle changes have been recommended to reduce the morbidity and mortality associated with hypertension [7]. Nevertheless, the cost, high dosing frequency and side effects are factors that affect patients' adherence with drug therapies, often undermining the drug efficacy [8, 9], making the use of non‐pharmacological treatment as an adjunct to usual care even more attractive [10].

Voluntary slow breathing exercise (VSBE) is defined as a breathing practice in which breathing‐related muscles are engaged in a specific sequence to carry out deep inhalation and exhalation at a rate of approximately six breaths per minute [11], which has gradually attracted attention in recent years as a potential non‐invasive method for modulating vagal activity [12, 13]. Several observational studies have demonstrated its effectiveness in lowering blood pressure [14, 15]. And studies have shown that variations in breathing frequency are closely associated with the autonomic nerve function [16, 17]. Slow and regular breathing can enhance baroreflex sensitivity and thus mitigate autonomic imbalance, a condition widely regarded as a significant contributor to the development of hypertension [18, 19]. Moreover, slow breathing may inhibit sympathetic output by reducing end‐tidal carbon dioxide and activating pulmonary stretch receptors [20], and this process may also indirectly enhance parasympathetic nerve activity, potentially reducing heart rate and blood pressure. Furthermore, deep and slow breathing also boosts the vascular blood flow in the small‐sized vessels and decreases the peripheral vascular resistance, contributing to a reduction in blood pressure [21]. These multi‐pathway effects collectively support VSBE as a promising non‐pharmacological strategy for hypertension management.

Currently, several meta‐analyses [22, 23] have evaluated the effects of slow‐breathing exercises, encompassing both device‐guided and non‐device‐guided approaches. To our knowledge, none has specifically focused on the outcomes of voluntary slow breathing exercise among individuals suffering from hypertension. Given the growing number of relevant RCTs, we conducted this meta‐analysis to focus on the effects of voluntary slow breathing exercise on cardiovascular parameters of hypertensive patients, aiming to provide theoretical support for healthcare providers in formulating optimal treatment strategies.

## 2 Materials and Methods

### 2.1 Design

This meta‐analysis was conducted following the Preferred Reporting Items for Systematic Reviews and Meta‐analysis (PRISMA) [24]. The protocol was registered on PROSPERO (CRD420251026840).

### 2.2 Data Sources and Literature Search

We employed a comprehensive search strategy integrating Medical Subject Headings (MeSH) and keyword combinations to systematically retrieve eligible literature from four databases: PubMed, Web of Science, Embase, and the Cochrane Library. The search span covered from the inception of each database to February 2025. Additionally, we conducted backward citation tracking by reviewing reference lists of relevant systematic reviews to identify potentially undiscovered studies, ensuring a thorough and unbiased evidence acquisition process.

### 2.3 Inclusion and Exclusion Criteria

Selection criteria were meticulously delineated following the “PICOS” framework: (1) Population: Hypertensive patients; (2) Intervention: Voluntary slow breathing exercise; (3) Comparison: Usual care, respiration at a normal rate, blank control, read or listen to music; (4) Outcomes: Systolic blood pressure, diastolic blood pressure and heart rate; (5) Study design: Randomized Controlled Trial (RCT).

The exclusion criteria are as follows: Conference abstract, randomized cross‐over studies, non‐English literature, device‐guided breathing, non‐RCT, the required information cannot be obtained or the full text cannot be found.

### 2.4 Data Extraction

The search results retrieved from online databases were transferred to EndNote 21, where duplicate literatures were systematically removed. Then two reviewers independently conducted the abstract screening and full‐text evaluation of the remaining articles following the pre‐set inclusion criteria. Subsequently, two reviewers extracted data from the eligible literatures into an Excel spreadsheet, comprising the publication year, country, study population, intervention parameters, control approach, as well as the outcome indicators. In case of any discrepancies, a third researcher will be involved in the decision‐making process.

### 2.5 Methodological Quality Appraisal and Risk of Bias

Two researchers independently appraised the methodological quality of eligible articles in accordance with the Cochrane Collaboration's risk‐of‐bias tool (Rob2) [25], which assesses seven domains: random sequence generation, allocation concealment, blinding of participants and personnel, blinding of outcome assessment, incomplete outcome data, selective reporting and other biases. If there were any disagreements between the two reviewers, a third reviewer was consulted to help reach a resolution when necessary. Any study that is determined to have low methodological quality will be excluded from the review. Finally, the risk of bias graph was constructed.

### 2.6 Data Analysis

Data analysis was performed using revman5.4 software. The comprehensive outcome index for continuous variables was presented as mean differences (MDs). We calculated the not available standard deviation (SD) based on the given CI limits, median and interquartile ranges. In the final analysis, the pre‐and post‐intervention differences were meticulously calculated to facilitate a comprehensive assessment. The I² statistic analysis along with the Χ² test will be performed to evaluate the possible heterogeneity existing among these studies. Statistical heterogeneity was deemed significant when the *p*‐value was ≤ 0.10 and the I² statistic was > 50%, prompting the adoption of a random‐effects (RE) model (Systolic blood pressure, Diastolic blood pressure, Heart rate). In contrast, when the heterogeneity was negligible, a fixed‐effects (FE) model was employed (LF/HF ratio). Subgroup analyses and sensitivity analyses will be performed if necessary.

## 3 Results

### 3.1 Search Results and Study Characteristics

**Figure 1:**
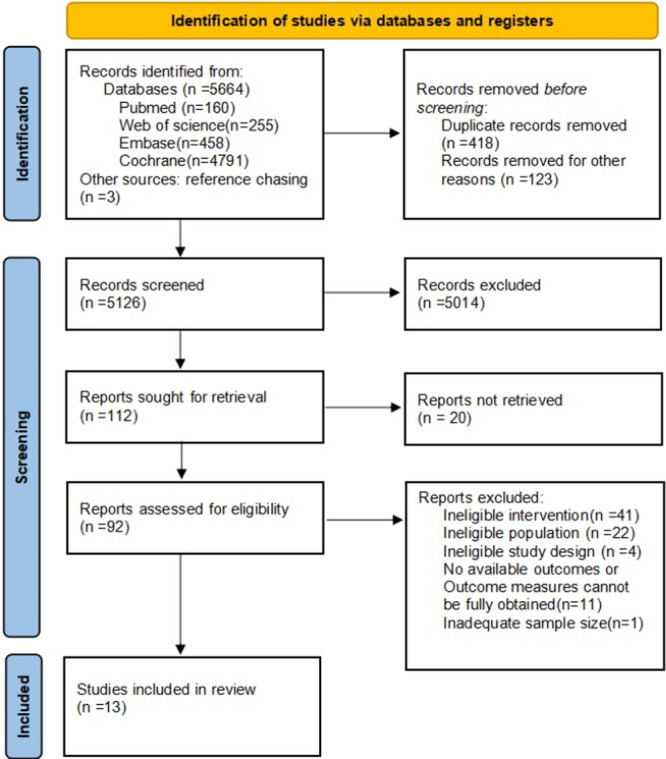
PRISMA Flow Diagram of Literatures Search and Included Studies for Systematic Review and Meta‐Analysis.

A total of 5,667 articles were initially retrieved from four databases (PubMed, Web of Science, Embase, and the Cochrane Library) via a comprehensive search strategy. Following a rigorous multi‐stage screening process, thirteen studies [26, 27, 28, 29, 30, 31, 32, 33, 34, 35, 36, 37, 38] involving 1,097 participants were ultimately included for quantitative analysis. Literature screening process is shown in Figure 1, and the basic characteristics of the included trials are presented in Table 1.

**Table 1:** Basic characteristics of the included studies.

Study	Country	Population	Intervention	Control	Duration	Outcomes
Yan Zou 2022	China	Essential hypertension Stage I or II	Slow breathing exercise at a rate of 4–6 breaths per minute	Usual care	12 weeks	①②③
Patel 1985	England	Hypertension	15–20 min of relaxation and meditation twice daily.	Routine health education	8 weeks	①②
Ubolsakka 2017	Thailand	Mild‐to‐moderate isolated systolic hypertension	30 min of slow‐loaded breathing training daily	Blank control	8 weeks	①②③
Mitsungnern 2021	India	Hypertensive emergency	Pursed‐lip breathing	Usual care	3 h	①②③
Márquez 2019	Spain	Blood pressure above normal or stage 1 hypertension	Mindfulness meditation, 2‐h group sessions per week plus 45‐min home practice per day	Health education	8 weeks	①②
Shetty 2017	India	Prehypertension or stage 1 hypertension	10‐min daily practice of Sheetali and Sheetkari pranayamas each	20‐min daily quiet sitting	1 month	①③④
Thanalakshmi 2020	India	Essential hypertension	30‐min daily Sheetali pranayama for 4 weeks	Blank control	4 weeks	①②③④
Cramer 2018	Germany	Primary arterial hypertension	90 min of yoga breathing exercises per week	Blank control	12 weeks	①②③
Modesti 2010	Italy	Essential hypertension	Daily 30 min slow breathing training at 4–6 breaths/min with 1:2 inspiratory‐expiratory ratio.	Read/listen to music	6 months	①②③
Srinivasan 2019	India	Pre‐ and stage1 hypertension	Practice for 30 min at 6 breaths per minute	Blank control	30 min	①②③
Misra 2019	America	Uncontrolled hypertension	Practice pranayama breathing at least five times weekly	Usual care	6 weeks	①
Sundaram 2012	India	Essential hypertension	Slow breathing twice a week in addition to conventional pharmacological treatment	Conventional pharmacological treatment	4 weeks	①②③
Ghati 2020	India	Essential hypertension	Bee‐humming breathing at a rate of 4 to 6 breaths per minute	Placebo control	5 min	①②③④

*Note:* ①Systolic blood pressure, ②Diastolic blood pressure, ③Heart rate, ④LF/HF ratio.

### 3.2 Risk‐Of‐Bias Assessment of the Included Studies

**Figure 2:**
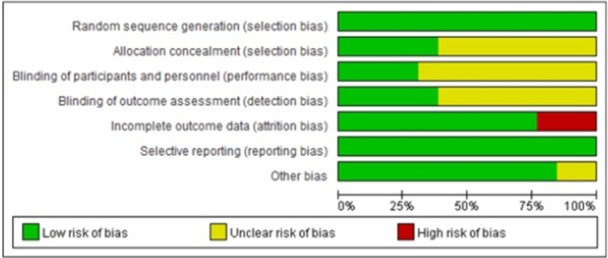
Risk of bias graph.

**Figure 3:**
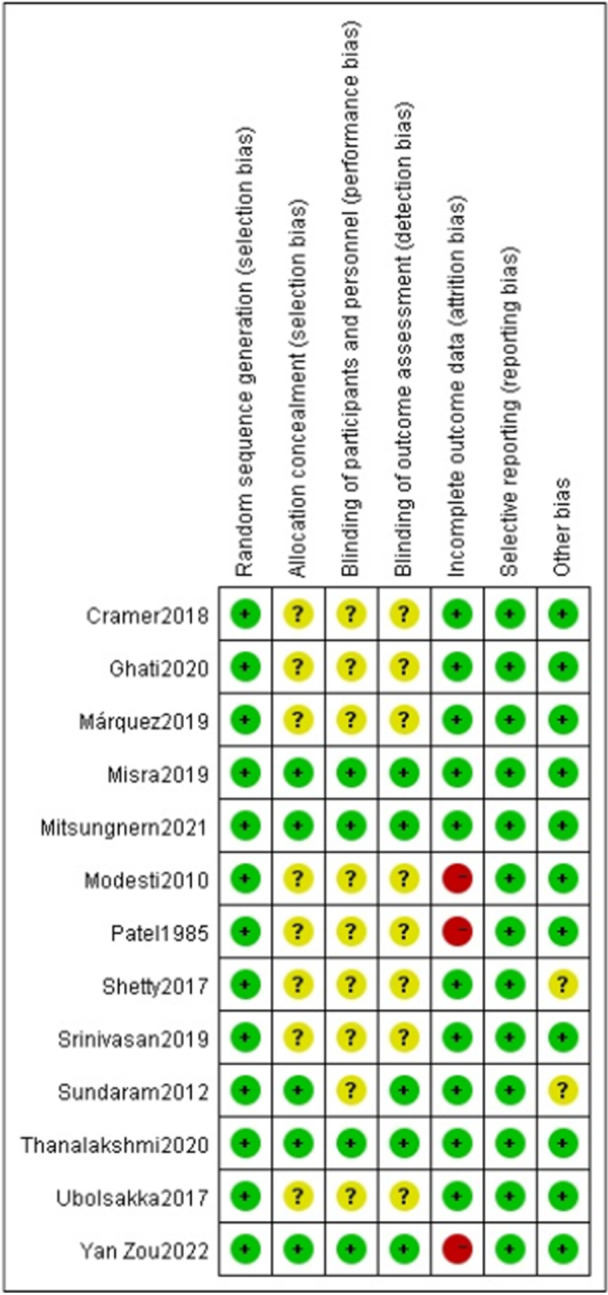
Risk of bias summary.

In terms of random sequence generation, all included studies [26, 27, 28, 29, 30, 31, 32, 33, 34, 35, 36, 37, 38] adopted random allocation methods and were assessed as having a low risk of bias. Five studies [26, 29, 32, 36, 37] were assessed as having a low risk of bias in allocation concealment, whereas eight studies [27, 28, 30, 31, 33, 34, 35, 38] did not explicitly report it. Regarding blinding, four studies [26, 29, 32, 36] employed a blinding design in their implementation and were therefore assessed as having a low risk of bias, while five studies [26, 29, 32, 36, 37] demonstrated low risk in relation to blinding of study outcomes. Three studies [26, 27, 34] exhibited high risk of attrition bias due to incompleteness of data. None of the studies showed selective reporting. Regarding other bias, two studies [31, 37] failed to describe the baseline comparability between the CHO group and control groups. The detailed risk of bias assessment and risk of bias summary were presented in Figure 2 and Figure 3.

### 3.3 The Outcomes

#### 3.3.1 Systolic Blood Pressure

**Figure 4:**
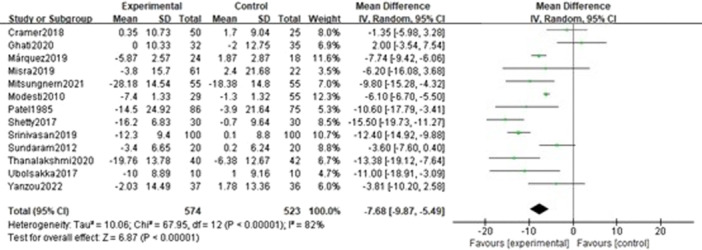
Forest plot of the effect of VSBE on systolic blood pressure.

Thirteen studies [26, 27, 28, 29, 30, 31, 32, 33, 34, 35, 36, 37, 38] appraised the changes in systolic blood pressure between the VSBE and control groups. Given the significant statistical heterogeneity (*p* < 0.001, I² = 82%) among the trials, a random‐effects model was adopted. The result revealed a noticeable mitigation (MD = −7.68; 95% CI = −9.87, −5.49; *p* < 0.001) in the VSBE group. More detailed results are presented in Figure 4.

**Figure 5:**
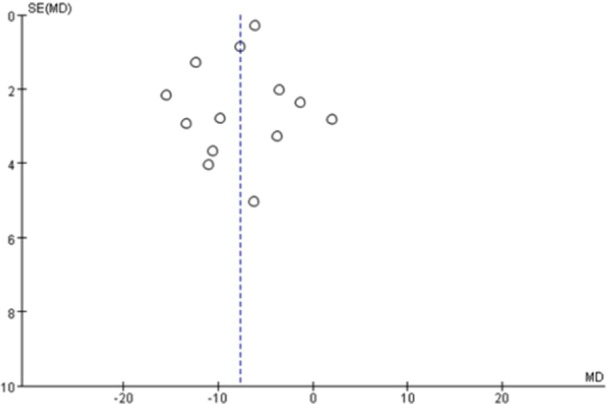
Publication bias funnel plot in systolic blood pressure.

A funnel plot was constructed to systematically assess publication bias, given the inclusion of more than 10 studies in the meta‐analysis (Figure 5).

#### 3.3.2 Sensitivity Analysis

The combined effect value did not change sequentially after sequentially excluding each study, indicating that the outcome was relatively stationary.

#### 3.3.3 Diastolic Blood Pressure

**Figure 6:**
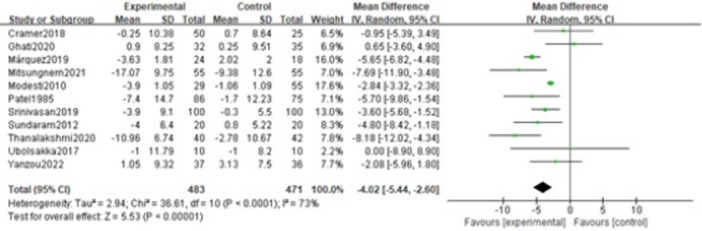
Forest plot of the effect of VSBE on diastolic blood pressure.

Diastolic blood pressure involved analysis of eleven studies [26, 27, 28, 29, 30, 32, 33, 34, 35, 37, 38]. Significant heterogeneity was observed among studies (*p *< 0.0001, I² = 73%), prompting a random‐effects model. The pooled analysis revealed that voluntary slow breathing exercise was significantly effective in reducing diastolic blood pressure compared with the control group (MD = −4.02; 95% CI = −5.44,−2.60; *p* < 0.001). More detailed results are shown in Figure 6.

#### 3.3.4 Heart Rate

**Figure 7:**
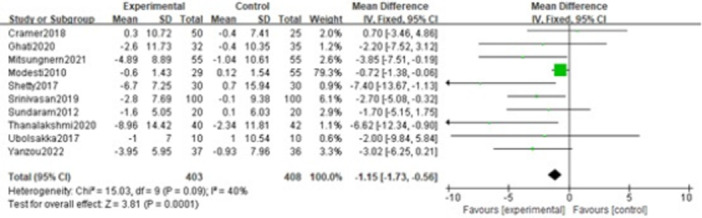
Forest plot of the effect of VSBE on heart rate.

In total ten trials [26, 28, 29, 31, 32, 33, 34, 35, 37, 38] evaluated the changes in heart rate between the intervention and control groups. A fixed‐effects model was performed to combine the effect size in view of no conspicuous heterogeneity (*p* = 0.09, I² = 40%) among the studies. The result showed that the voluntary slow breathing exercise group had an apparent decrease (MD = −1.15; 95% CI = −1.73, −0.56; *p* < 0.001) in heart rate compared to the control group. More detailed results are presented in Figure 7.

#### 3.3.5 LF/HF Ratio

**Figure 8:**
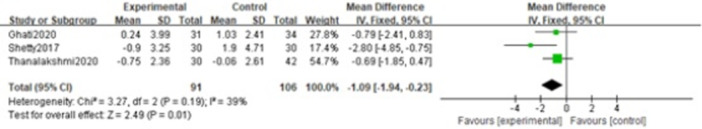
Forest plot of the effect of VSBE on LF/HF.

Only three studies [31, 32, 38] assess the changes in LH/HF ratio. The lack of significant heterogeneity (*p* = 0.19, I² = 39%) among the studies prompted us to use the fixed‐effects model. The results indicated that slow breathing exercise had a certain beneficial effect (MD = −1.09; 95% CI = −1.94, −0.23; *p* = 0.01) on the LF/HF ratio. The specific results are shown in Figure 8.

Sensitivity analysis using the leave‐one‐out method revealed fluctuations in the effect size of the results, suggesting a potential instability in the findings.

## 4 Discussion

The findings of this meta‐analysis demonstrate that voluntary slow breathing exercise is an effective adjunctive intervention, producing clinically meaningful cardiovascular benefits in hypertensive patients. The observed reductions in blood pressure and heart rate are not only statistically significant but also clinically important, as they translate to substantially lower risks of stroke and cardiovascular mortality. Beyond confirming efficacy, this study reveals that longer intervention durations do not necessarily confer greater benefits, underscoring the importance of identifying the optimal duration and intensity balance in future research. These results position voluntary slow breathing as a viable, low‐cost adjunct to pharmacological therapy, aligning with the 2023 WHO Guidelines for the Management of Hypertension, which recommend low‐cost non‐pharmacological interventions as a first‐line approach for patients with mild hypertension [6].

The integrated data from thirteen randomized controlled trials (RCTs) involving 1,097 patients with hypertension demonstrated that voluntary slow breathing exercise can significantly decrease systolic blood pressure (MD = −7.68; 95% CI = −9.87, −5.49; *p* < 0.001), diastolic blood pressure (MD = −4.02; 95% CI = −5.44,−2.60; *p* < 0.001), heart rate (MD = −1.15; 95% CI = −1.73, −0.56; *p* < 0.001) and LF/HF ratio (MD = −1.09; 95% CI = −1.94, −0.23; *p* = 0.01). This result is consistent with a meta‐analysis by [22] but with a further focus on hypertensive populations. The findings of this study are of significant clinical importance, as it has been demonstrated that moderate reductions in blood pressure and heart rate can decrease the risk of cardiovascular events. Sundstrom et al. [39] found that, a mean blood pressure reduction of 3.6/2.4 mmHg was associated with a 28% reduction in stroke risk and a 25% reduction in cardiovascular death risk. Data from 4,065 patients revealed that each one‐beat‐per‐minute change in heart rate was associated with a 1% alteration in mortality risk among hypertensive patients [40]. Based on these findings, as a non‐pharmacological intervention, voluntary slow breathing exercise may confer cardiovascular benefits to patients with hypertension, particularly those with mild‐to‐moderate hypertension or individuals seeking to reduce the medication burden. A meta‐analysis conducted by [41] found a trend toward greater reduction with increasing intensity, suggesting the intensity of the intervention could certainly play a role in the level of blood pressure reduction. They found that interventions with higher weekly minutes were associated with greater reductions in both systolic and diastolic blood pressure. For instance, interventions lasting less than 100 min per week resulted in a modest SBP reduction of −3.01 mmHg, while those lasting between 100 and 200 min per week led to a more pronounced reduction of −6.44 mmHg. The most intensive intervention, exceeding 200 min per week, was associated with the largest decrease in SBP (−14.00 mmHg), although this finding was based on a single trial and should be interpreted with caution. This dose‐response relationship suggests that the physiological benefits of slow breathing may be cumulative, potentially mediated by enhanced autonomic regulation, reduced sympathetic activity, and improved baroreflex sensitivity over time. Greater weekly exposure to slow breathing may lead to more sustained neurocardiovascular adaptations, thereby amplifying blood pressure‐lowering effects. However, the studies included in this meta‐analysis showed that, longer intervention durations were not consistently associated with greater efficacy, and in some cases, shorter interventions yielded comparable or superior outcomes. For instance, a 12‐week intervention study [33] demonstrated that its blood pressure‐lowering effect was paradoxically less significant than that of studies with shorter intervention durations (4 weeks) [31, 32]. This result may be attributed to declining participant adherence, adaptive physiological resistance, or differences in specific breathing exercise modalities. Future research should prioritize comparative RCTs of different breathing exercise protocols to inform standardized regimens for hypertensive patients. Concurrently, efforts should focus on establishing optimal dosing parameters and exploring whether a threshold effect exists beyond which additional intensity yields diminishing returns.

With respect to mechanisms, slow breathing primarily influences heart rate and blood pressure through the following pathways. Firstly, the core feature of hypertension is autonomic nervous system imbalance, and the key mechanism underlying this imbalance lies in reduced arterial baroreflex sensitivity and enhanced chemoreflex activation [42, 43]. By reducing respiratory frequency, slow breathing can specifically enhance arterial baroreflex sensitivity [44] and optimize cardiovascular reflex regulation, thereby alleviating autonomic imbalance and reducing blood pressure. Moreover, respiratory frequency influences respiration‐induced sympathetic nerve activity and peripheral resistance regulation. When the respiratory frequency is reduced to 6–10 breaths per minute, tidal volume increases, which in turn induces stimulation of cardiopulmonary stretch receptors [45]. This attenuates sympathetic nerve discharge, which in turn elicits a decrease in systemic vascular resistance and subsequent lowering of both blood pressure and heart rate through a feedback‐mediated mechanism [46].

Heart rate variability (HRV) has been widely used as a non‐invasive indicator to assess the function of the autonomic nervous system (ANS), which allows the detection of two main periodic components through spectral analysis of continuous electrocardiogram (ECG) signals: low frequency (LF) and high frequency (HF) [47]. The LF/HF ratio serves as the core indicator in heart rate variability (HRV) analysis, directly reflecting the balance of sympathetic‐parasympathetic neural tone. In this meta‐analysis, only three studies assessed the effect of slow breathing exercises on LF/HF ratio. Although the initial meta‐analysis showed a significant effect (MD = −1.09; 95% CI = −1.94, −0.23; *p* = 0.01), the leave‐one‐out sensitivity analysis revealed instability in the results. The instability of the results may be attributed to the limited number of studies, heterogeneity of interventions, and the HRV measurement methods. Owing to the limited number of studies and the noted instability in sensitivity analysis, the existing evidence regarding the LF/HF ratio warrants cautious interpretation. This also underscores the need for rigorous, large‐scale studies to strengthen the reliability of conclusions in this area.

This study supports the integration of voluntary slow breathing exercises into comprehensive management protocols for hypertensive patients, particularly suitable for those with mild‐to‐moderate hypertension or drug intolerance. Critical considerations for clinical implementation include: Ensure patients master appropriate breathing frequency and rhythm through standardized tutorials such as video guidance and APP‐based monitoring systems. It is recommended that they be used synergistically with pharmacotherapy and exercise interventions to develop multidimensional lifestyle management strategies. It is advised to improve the exercise compliance of patients through telephone follow‐up to improve the persistence of the intervention and increase the intervention effect.

## 5 Limitations

This meta‐analysis had several limitations. Firstly, despite rigorous literature screening guided by predefined inclusion criteria, notable heterogeneity persisted in the design of intervention protocols across included studies. For example, variability was evident in several dimensions, including the specific form of slow‐breathing practices such as mindfulness meditation and relaxation [27, 30], yoga breathing [33], pursed‐lip breathing [29], and pranayama breathing [31, 32, 36], and duration of intervention (ranging from 30 min to 6 months). Furthermore, the included studies spanned various countries including the America [36], India [29, 31, 35, 37, 38], China [26], Spain [30], Thailand [28], Germany [33], Italy [34], and England [27]. Such heterogeneity may have attenuated the pooled effect size of the meta‐analysis. However, broad geographical coverage suggests the effectiveness of intervention is generalizable to hypertensive patients worldwide, regardless of local healthcare contexts or cultural backgrounds, strengthening the external validity of the meta‐analysis results. Future investigations should prioritize the development of standardized slow‐breathing regimens with uniform parameters to mitigate intervention‐related biases. Moreover, although this study has confirmed the association between slow breathing and improvements in blood pressure and heart rate, underlying mechanisms such as vagal nerve activation and endothelial function improvement have been sparsely addressed in a few studies, and there is a lack of evidence from biomarkers. Future research should incorporate monitoring of physiological indices such as heart rate variability and hemodynamic parameters to deeply elucidate the blood pressure‐lowering mechanisms of slow breathing exercises.

## 6 Conclusion

In conclusion, voluntary slow‐breathing exercise demonstrates significant improvements in blood pressure and heart rate among hypertensive individuals, serving as a viable non‐pharmacological intervention for hypertension.

## Author Contributions

ShenTing CHENG's contributions were the conception of the study and manuscript writing. Yan CHEN drafted the manuscript. Man YU and LiShuang ZHAO extracted the data, evaluated the quality and analyzed the data. Hui HUANG and MinYan ZHAO conducted a rigorous review and revision of the manuscript. All the authors have read and agreed the published version of the manuscript.

## Funding

The authors received no specific funding for this work.

## Conflicts of Interest

The authors declare no conflicts of interest.

## Data Availability

The authors have nothing to report.
